# Function and dysfunction of plasma cells in intestine

**DOI:** 10.1186/s13578-019-0288-9

**Published:** 2019-03-13

**Authors:** Xue Wang, Gui-liang Hao, Bo-ya Wang, Chen-chen Gao, Yue-xiu Wang, Li-sheng Li, Jing-dong Xu

**Affiliations:** 1 School of Basic Medical Sciences, Xuanwu Hospital, Beijing Capital Medical University, Beijing, 100069 China; 2 Peking University Health Science Center, Beijing, 100081 China; 3 Department of Physiology and Pathophysiology, School of Basic Medical Science, Capital Medical University, No. 10, Xitoutiao, Youanmenwai, Fengtai District, Beijing, 100069 China; 4 Department of Teaching Office, International School, Capital Medical University, Beijing, 100069 China; 5 Function Platform Center, School of Basic Medical Science, Capital Medical University, Beijing, 100069 China

**Keywords:** Plasma cells, Antibody, Immunomodulation, Intestine, Inflammatory bowel disease, Food allergy, Tumors

## Abstract

As the main player in humoral immunity, antibodies play indispensable roles in the body’s immune system. Plasma cells (PCs), as antibody factories, are important contributors to humoral immunity. PCs, recognized by their unique marker CD138, are always discovered in the medullary cords of spleen and lymph nodes and in bone marrow and mucosal lymphoid tissue. This article will review the origin and differentiation of PCs, characteristics of short- and long-lived PCs, and the secretion of antibodies, such as IgA, IgM, and IgG. PCs play a crucial role in the maintenance of intestinal homeostasis using immunomodulation though complex mechanisms. Clearly, PCs play functional roles in maintaining intestinal health, but more details are needed to fully understand all the other effects of intestinal PCs.

## Core tip

This manuscript has the following components. First, after describing the origin of plasma cells (PCs), it summarizes their general biological features and common functions. The second component reveals the characteristics of short-lived PCs (SLPCs) and long-lived PCs (LLPCs). Notably, we depict how LLPCs survive in bone marrow and why they could be a double-edged sword in the human body. Thirdly, we summarize the functions of PCs, especially their function in the intestine. Next, we address some relationship between PC disfunction and intestinal diseases. Last but not least, some novel viewpoints on the pathogenesis of inflammatory bowel disease (IBD) and the unique role of IgA in intestinal food allergies and tumors are also emphasized.

## Introduction

The gastrointestinal mucosa contains numerous plasma cells (PCs) under normal condition. Immunocytochemical studies [1] have shown that most of these PCs contain (and presumably produce) immunoglobulin A (IgA). Immunoglobulin G and M (IgG and IgM, respectively) can be detected in a few of these PCs. Tolerance in the intestinal immune system is required to inhibit immunity against commensal bacteria, as well as an array of dietary antigens. The microenvironment regulates PC survival in the small intestine [2]. Additionally, bacterial Toll-like receptor (TLR) ligands induce survival factors of PCs, such as a proliferation-inducing ligand from intestinal epithelial cells [3], lamina propria dendritic cells (LP-DCs) [4], and mucosal neutrophils [5]. Bacterial exposure induces not only LP-PC survival but also the generation of specific IgA-secreting (IgA+) LP-PCs [6]. We found that the number of IgA+ PCs was substantially increased in other immune organs, such as Peyer’s patches (PPs) [7], mesenteric lymph nodes [7–9] (MLNs), spleen [10], and bone marrow (BM) [11], from colony-transferred mice. IgA+ B cells in the PPs, MLNs, and spleen are considered the origin of IgA+ LP-PCs [7]. However, the IgA+ cells detected in these organs after the colony transfer are also CD138+, indicating that they are already differentiated PCs. Numerous Ig molecules are secreted by PCs. With the background described above, it seems obvious that intestinal PCs play a unique supporting role in maintaining the balance of intestinal immunity, and they are more complex than initially thought.

In this review, after briefly describing the origin of PCs, we summarize their general biologic features and common functions to survey the differences between PCs in the intestine and other tissues. Notably, we review how PCs participate in maintaining intestinal health or disorder with PC dysfunction. To further indicate the regulatory function of PCs in intestine, we review the latest progress concerning tumors that have been attributed to shortcomings of immunological surveillance and immunoregulation. The colonic immune system, created by the innate and the acquired immune systems in a coordinated manner, has unique adaptations to limit both microbial exposure and immune responses, ensuring a limited response to commensal organisms under normal conditions [12, 13]. Any dysfunctions occurring in components of immune system can significantly disrupt colonic homeostasis.

Every B cell is specific to a single antigen, but each cell can produce several thousand matching antibodies per second [14]. This prolific production of antibodies is an integral part of the humoral immune response. Furthermore, some promising targets are emerging for the study and treatment of IBD or tumors. However, more findings unearth more puzzles, and many details of intestinal PCs and the mechanistic understanding for IBD and intestinal allergy remain obscure and are discussed in the manuscript.

## Occurrence of PCs

In 1963, Max D. Cooper and others discovered that chicken bursa of Fabricius could produce antibodies when they were investigating immune defense mechanisms, opening the era of B cell and PC research. PCs, also called effector B cells, are antibody factories that can synthesize and secrete thousands of clone-specific antibody molecules per second. Therefore, they play a crucial role in the intestinal immune system and are required to inhibit immunity against commensal bacteria, as well as an array of dietary antigens. PCs are the fully differentiated cells of the B-cell lineage [15] under physiological or pathophysiological conditions. Human B cells, derived from pluripotent stem cells of fetal liver or BM through pre-B cells, immature B cells, and eventually differentiated mature B cells, are transported to the lymph nodes or spleen via the blood circulation and exist in the lymphoid nodules of the lymph node cortex area, medullary cord of the medullary area, perisplenic region and acini lienalis.

Cytokines [interleukin (IL)-2, IL-6, and IL-10] and direct cell-to-cell contact between T and B cells play vital roles in B-cell differentiation to PCs. In the lymph nodes, naïve follicular B cells, located in B-cell follicles, are surrounded by T-cell zones. After encountering an exogenous antigen, B cells proliferate and migrate to the border of the T-cell zone or interfollicular region, in which they interact with antigen-specific CD4+ T cells to become fully activated.

Differentiation of B cells into PCs is affected by external and internal factors. Activated B cells can express IL-6 receptors, which can induce the differentiation to PCs [16, 17]. However, IL-6 alone cannot promote the differentiation to PCs [18]. IL-10 also functions to increase PCs. IL-10, originally identified as a TH2 helper T-cell product, can inhibit cytokine production by TH1 cells, but it induces PCs to secrete large amounts of IgG, IgA, and IgM [19, 20]. Furthermore, the interactions between CD27, which belongs to the nerve growth factor receptor/tumor necrosis factor receptor (NGFR/TNFR) family [21], and CD27 ligand (CD70), which belongs to the TNF family [22], occur not only on activated memory B cells but also on T cells, especially activated CD41 CD45RO T cells [23], thus enhancing Ig production by B cells [24, 25] and suggesting that the CD27/CD70 interaction is involved in the differentiation of CD27+ memory B cells into PCs. These cell-surface molecules promoting the differentiation of B cells cooperate with each other to complete the differentiation of B cells into PCs by complex signaling pathways.

Other activities within the cell promote the differentiation of B cells into PCs. Over the past two decades, a handful of master regulators of PC differentiation have been identified. These transcription factors include B lymphocyte-induced maturation protein-1 (BLIMP-1), interferon regulatory factor 4 (IRF4), and X-box binding protein-1 (XBP-1) [26–32]. In 1994, Turner [33] identified BLIMP-1, a 98-kD protein containing five zinc finger new structures, which is involved in the deacetylation and methylation of histone. All PCs and a small group of germinal center (GC) B cells can express BLIMP-1, but it is noteworthy that BLIMP-1 cannot be expressed by naïve or activated B cells. The differentiation process is accompanied by the expression of *Blimp*-*1*, indicating that *Blimp*-*1* mainly may play a fatal role in the differentiation and development of B cells in the GCs. However, whether BLIMP-1 is involved in the differentiation of B cells into PCs is ambiguous. In 2003, Shapiro-Shelef et al. [34] summarized the experience of a previous investigation that failed to study BLIMP-1-deficient mice and then skillfully devised a prdm1 flox/floxCD19Cre/+ mouse. Using NP-FICOLL (TI-antigen) and NP-KLH (TD-antigen) to stimulate the mice, they found that antigen-specific amplification does not depend on BLIMP-1 [35], but the presence of short-lived PCs (SLPCs) and long-lived PCs (LLPCs) produced by germinal centers requires the participation of BLIMP-1. At the same time, intraperitoneal injection of tamoxifen to remove the *prdm1* gene in the BM in vivo was used to observe the number of PCs, and the activation of B cells with LPS was used to observe alteration of the CD138+ PC level in vitro, confirming that BLIMP-1 is required for PC maintenance.

*Blimp*-*1* induces PC development through at least three gene expression programs. First, *Blimp*-*1* blocks the hyperplastic procedure of B cells, such as direct inhibition of *c*-*myc* [36]. Second, Blimp-1 can upregulate some genes that promote Ig secretion, such as Ig heavy and light chain genes, J chain, XBP-1, C/EBP homologous protein (CHOP), and HSP70. Finally, *Blimp*-*1* downregulates other genes that play important roles in the formation of the germinal center and B-cell activation, such as Pax-5 [37], Bcl-6, activation-induced cytidine deaminase (AID), BCR signaling-related genes, CD72, and CXCR5. If any of the three gene expression programs is disrupted, disease may occur, such as autoimmune diseases [38–42]. Therefore, there is a tremendous need to study the mechanism of *Blimp*-*1* in PCs differentiation.

Additionally, BLIMP-1 is affected by multiple regulatory pathways [43]. The B cell-specific coactivator OBF-1 was found to be a positive regulator of BLIMP-1 by means of OBF-1 knockout mice compared with the wild-type (WT) mice [44]. In BLIMP-1 activation, the extracellular signal-regulated MAP kinase/mitogen-activated protein kinase (ERK/MAPK) pathway was discovered to be another important pathway using conditional ERK2-knockout mice [45]. Moreover, conditional v-Rel avian reticuloendotheliosis viral oncogene homolog A (RelA) knockout mice showed that the nuclear factor kappa B (NF-κB) pathway is also significant in BLIMP-1 regulation [46]. Above all, BLIMP-1 can play an indispensable role in PCs differentiation.

IRF4, as essential for class switch transformation (CSR) and PC differentiation [47–49]. IRF4 appears to positively regulate BLIMP-1; without it, BLIMP-1-mediated PC differentiation does not proceed [49]. Moreover, IRF4 and STAT3 activate BLIMP-1 in the late GC/early PB stages of PCs differentiation [30]. However, in recent years, some contrasting research found that IRF4 is dispensable in B cells for GC development, while others demonstrated that it is indispensable in B cells for GC formation by RNA-Seq analysis in ex vivo-derived mice [26, 31]. Nevertheless, IRF4 is required for GC formation and differentiation into PCs; however, the exact role of IRF4 in GC formation and whether B or T cells are involved in the intrinsic mechanism remain obscure.

Meanwhile, XBP-1, a component of the unfolded protein response (UPR), also plays an indispensable role in the differentiation of PCs. Relieving endoplasmic reticulum (ER) stress is the main function of UPR [50]. The protein kinase RNA activated (PKR)-like ER kinase (PERK), activating transcription factor 6 (ATF6a), and inositol-requiring enzyme-1 (IRE1) activate a myriad of factors from chaperone proteins to protein trafficking proteins to calcium modulators and, if necessary, apoptosis proteins [51]. Upon antigen stimulation, B cells differentiate into antibody-secreting cells (ASCs), which requires expansion of the ER and XBP1. Moreover, normal and malignant ASCs are exquisitely sensitive to proteasome inhibitors, however the underlying mechanisms remain unclear. CHOP, which mediates apoptosis in lots of cell types, expresses at high-level under ER stress. Unlike other cell types, *chop*^−*/*−^ ASCs are slightly more sensitive to ER stressors and proteasome inhibitors, signifying tissue-specific roles for CHOP in differentiation and stress [52].

Likewise, as a transcription factor containing a bZIP domain, XBP-1 has been implicated as a required factor in the generation of ASCs over the last 20 years. XBP-1 is a component of the IRE1 branch of the UPR. IRE1 can yield active XBP-1 (XBP-1s) by splicing the transcript of XBP-1 [50]. A study using murine splenic cells revealed XBP-1 to be downstream of BLIMP-1 in the PC differentiation program [53]. Mice lacking XBP-1 showed lower serum Ig than WT mice [32]. Furthermore, XBP-1 was not required in initial IgM μ chain synthesis; nevertheless, a few days after activation, XBP-1 deficiency might result in a steep decrease in Ig production [54]. In contrast, XBP-1 had no effect on light chain synthesis [54]. Therefore, the need for XBP-1 has been demonstrated, and it is responsible for factors involved in the secretory machinery of these ASCs. Above all, XBP1 is essential for the differentiation of PCs. This differentiation requires not only the expression of XBP1 but also the spliced isoform XBP1s. XBP1 regulates PC differentiation independently via its known functions in the ER stress response [55]. there is abnormal expression of XBP1, IRF4 or Blimp1, all PC differentiation-related genes, there will be maladjustment because XBP1-deficient PCs will fail to colonize their long-lived niches in the BM and sustain antibody secretion.

## Characteristics of SLPCs and LLPCs

The first contact of B cells with an antigen leads to the formation of antibody-secreting PBs, which will live for less than 1 week in extrafollicular foci and result in a short antibody response—that is, primary immune responses generate short-lived proliferative PBs, producing a transient burst of antigen-specific antibodies. Nonetheless, most ASCs, including antibody-secreting PBs and PCs generated during the memory immune response, leave the follicles of secondary lymphoid tissues as PBs. The features of the ASCs influence the duration of the antibody response, in addition to the affinity and isotype of the antibody that is made, all of which comprise important characteristics of protective immunity.

Fragment crystallizable domain (Fc) receptor (FcR)-associated chain-null mice (*Fcγ*^−*/*−^) demonstrate a selective defect in the expression of FcRs on the cell surface and in the FcR-mediated signaling cascade [56], and surprisingly, *Fcγ*^−*/*−^ mice preferentially develop SLPCs and fail to develop a GC response. These results show that *Fcγ*^−*/*−^ mice display class switching to IgG2a as well as IgG1 and produce significantly higher serum titers of specific antibody than WT mice. Furthermore, *Fcγ*^−*/*−^ DCs show a more mature phenotype and produce at least two-fold more IL-12 than WT DCs. IL-12 can induce the formation of PCs and B-cell heavy chain class switching to IgG1 and IgG2a. The switch to IgG2 is IFN-ɣ dependent; however, it is not required for IFN-γ to promote the differentiation to PCs and switch to IgG1 [57]. Although IL-12 promotes SLPCs, perhaps in synergy with CD40L [57], memory B cell differentiation occurs independently of IL-12 [58, 59]. Therefore, IL-12 produced by DCs seems to be highly influential in directing B cells to differentiate into SLPCs. However, antibodies produced by SLPCs have not only a short lifespan but also a low affinity with antigens, which are easy to remove in autoimmune diseases. Thus, their characteristics bring convenience to treatment. However, the antibodies secreted by LLPCs have a long lifespan.

**Fig. 1:**
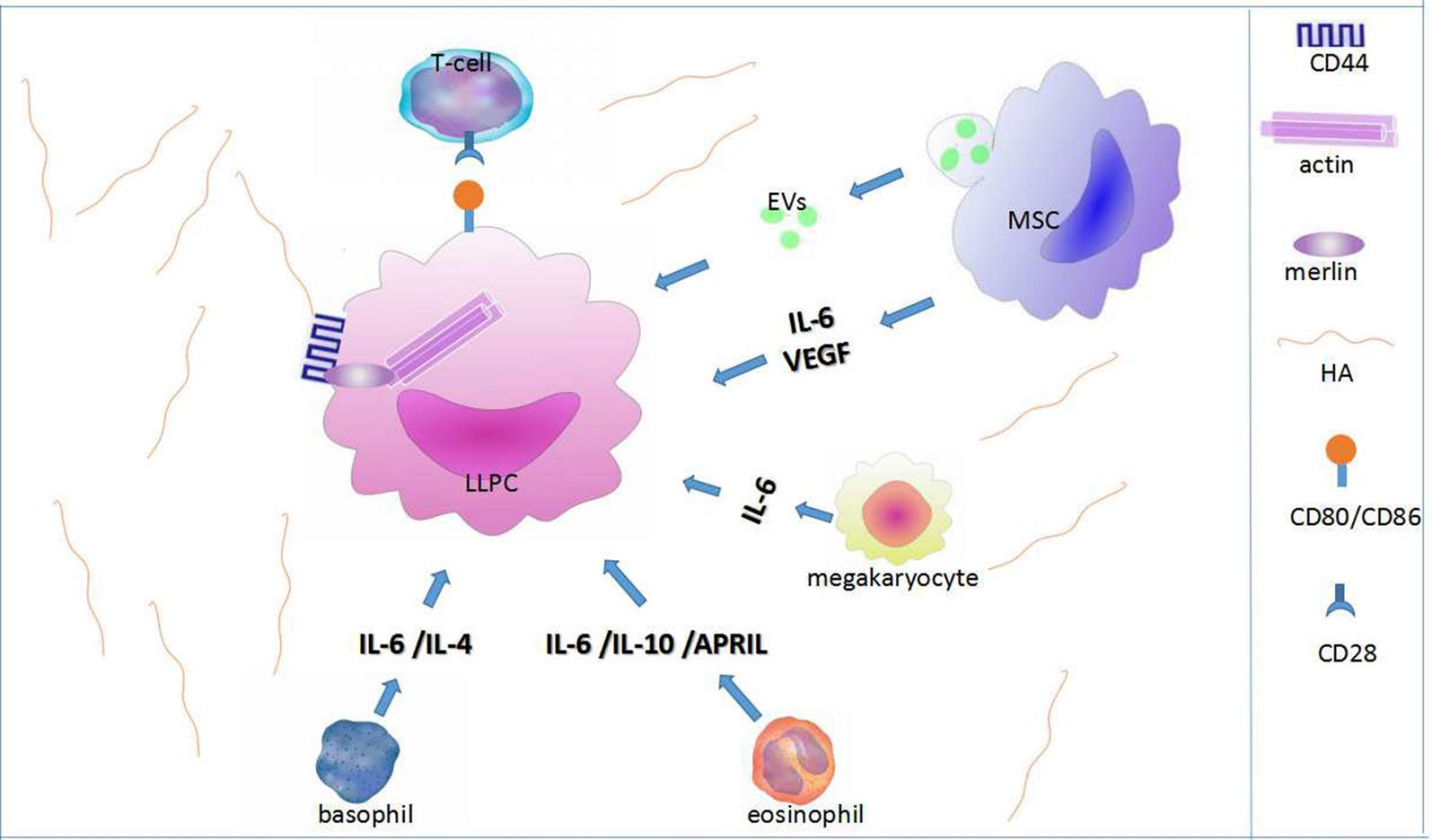
Survival mechanism of LLPCs in bone marrow. IL-6 and VEGF from MSC secretion mediate the survival mechanisms of the PCs in BM. Beyond that, some of the LLPC survival effects in BM can occur via MSC-derived EVs, such as exosomes and MVs. Basophils can positively affect PC Ig secretion by secreting IL-6 and IL-4. Activated eosinophils secrete some cytokines, including APRIL, IL-6 and IL-10. Megakaryocytes support PC growth and survival by producing large amounts of IL-6. Communication between CD28 and its ligands CD86 and CD80 can sustain the survival of LLPCs [93]. When the number of PCs is reduced, the extracellular portion of CD44, a transmembrane glycoprotein, binds to the HA of the ECM, while the intracellular portion of CD44 binds to actin through the phosphorylation of merlin to form a growth-promoting transfer complex. *MSC* mesenchymal stem cells, *HA* hyaluronic acid, *ECM* extracellular matrix, *merlin* ERM family member [196]

As mentioned above, during the secondary immune response, most of the ASCs generated leave the follicles of secondary lymphoid tissues as PBs. Although the human recombinant c-fragment of TT (rTT.C)-specific PBs expresses the corresponding chemokine receptors, CXCR3 and CXCR4, only at low levels, they migrate in response to chemokine (C-X-C motif) ligand 9 (CXCL9) and CXCL12. Attraction by CXCL9 and CXCL12 promotes the migration of recently generated CXCR3^low^/CXCR4^low^/CD95^intermediate^ MHC class II^high^ PBs into the BM or inflamed tissue [60–63]. The BM contains multiple microenvironmental niches that is appropriate for cellular proliferation, differentiation and survival [64–69]. Each niche seems to support specifically one or a few hematopoietic stem or precursor cells. In this way, the sizes of these populations are limited by the number of available niches [65, 70]. Therefore, newly generated CXCR3^low^/CXCR4^low^/CD95^intermediate^ MHC class II^high^ PBs apparently compete with resident CXCR3^high^/CXCR4^high^/CD95^low^ MHC class II^low^ PCs for survival niches. Some authors have proposed that resident PCs from the BM are activated during an immune response. Because their numbers are nearly equivalent to the newly generated PBs, it is also thought that the BM-resident PCs are expelled from survival niches by newly generated PBs [71]. For mice, after immunization, BM-resident PCs occur late and do not migrate toward CXCL12, although resident PCs express high amounts of CXCR4. Specific ASCs are also found in BM [72–74], mucosa-associated lymphoid tissues, chronically inflamed tissues [75], and, to a lesser extent, the red pulp of spleens [76], which are mature PCs with a potential lifespan of more than 18 months [71, 72, 77]. Therefore, in mice, PCs of the BM can survive for a long time, nearly up to the lifespan of the immune system [72, 77], and this phenomenon can be inferred indirectly to occur in humans [78]. However, an increasing number of investigations have suggested that PC survival is not cell autonomous but depends on signals provided by its living environment. Benner et al. discovered that the most potent PC survival factors identified thus far are a proliferation-inducing ligand (APRIL), IL-6, TNF-α, stromal-derived factor-1α, and signals transduced via CD44 [79–82] (Fig. 1). Additionally, BM mesenchymal stem cells (MSCs) provide survival factors in the human BM microniche that support LLPC survival [83–86]. The survival mechanisms of the BM microniche are thought to be mediated by local paracrine MSC secretion of IL-6 and vascular endothelial growth factor (VEGF) [87–89]. Basophils can positively regulate PC Ig secretion by markedly increasing IL-6 [90]. Additionally, activated eosinophils can secrete some cytokines, including IL-6, APRIL and IL-10 [91]. BM PCs share a microniche with megakaryocytes that can produce large amounts of IL-6 to support PC growth and survival [92].

Paracrine signals from a series of cytokine-secreting cells mediate PC survival as well as Ig secretion, and there are direct cell–cell interactions that play significant roles in this process. One of the interactions is the communication between CD28 and its ligands CD86 and CD80. CD28 is expressed on PCs [93], as CD28^−/−^ mice have reductive serum Ig titers, and the reduction may be due to PC intrinsic signaling by CD28 and not to a deficiency in activating T cells [93]. Developing PCs and LLPCs of the BM express the surface marker CD93, while CD93^−/−^ mice exhibit impaired antibody production following immunization [94]. In additional, the latest investigation [95] has re-confirmed that PC survival effects in BM can occur via MSC-derived extracellular vesicles (EVs), such as exosomes and microvesicles (MVs). EVs, which released from various cell types that have features distinguished by tetraspanins on their membrane surface, such as CD9, CD63, and CD81. The proteomic assessment of EVs has noted the significance of the PLC signaling pathway, including the presence of signaling proteins, specifically ras-like proto-oncoproteins A (RALA) and B (RALB). These proteins are both guanosine triphosphatases (GTPases) and act in close association with G-protein-coupled receptors (GPCR) to transduce signaling events via GTP hydrolysis; Moreover, RALA is required to suppress apoptosis [96]. Thus, EVs, which derived from BM-MSC secretions, can enhance survival of human ASC and provide a new field to explore PCs survival in BM.

LLPCs have been proposed in humans to explain the persistence, in the absence of antigen-specific stimulation, of antimicrobial antibodies with very long half-lives that have high affinity to and can provide protection against previously encountered pathogens. These LLPCs are important for maintaining protective antibody memory. However, this typical of a feature is also a double-edged sword because autoimmune diseases are related to the differentiation and imbalance of LLPCs and SLPCs. So autoantibody-secreting LLPCs are refractory to conventional immunosuppressive therapy and represent a therapeutic challenge in autoimmune diseases [96–98].

## Function of PCs

### Antibody secretion

PCs, as white blood cells, can synthesize, integrate, and secrete large volumes of antibodies. Igs, essential molecules for a successful immune response to pathogens, can allow the immune system to recognize a myriad of pathogens and respond to them effectively [99]. Before exposure to antigen, a diverse antibody repertoire is generated early in the development of B cells through the successful rearrangement of the D, V, and J gene segments to create B cells. Each gene segment produces a particular Ig heavy- or light chain variable (V) region [99]. The light chain and heavy chain genes of Ig undergo recombination, leading to the specificity for a unique antigen [100].

Upon encountering antigen and subsequent activation, determined by the nature of the antigen, some activation of B cells occurs with the assistance of CD4+ TH cells [T-dependent activation (TD)] and without the assistance of TH cells [T-independent activation (TI)] [101]. Subsequently, B cells can not only differentiate into SLPCs but also enter GCs and differentiate into either LLPCs or memory B cells [101].

GCs are significant in Igs regulation because they are the location of somatic hypermutation (SHM), which can produce high-affinity antibodies, and one of the sites of class switch recombination (CSR), which change the functions of produced antibodies [102, 103]. SHM and CSR are largely targeted to the Ig genes. Regarding SHM, the mutations are mainly single-base substitutions, with occasional insertions and deletions. Although mutations occur throughout the rearranged V regions, there is preferential targeting to some hot spots which are WRCY (W = A or T, R = A or G, C, Y = T or C) and WA, suggesting that there are higher-order structures or other local sequences which may also affect the mutations targets [104, 105]. In CSR, the Cµ region in naïve B-cell Ig genes is replaced by downstream Cγ, Cε or Cα segments to generate IgG, IgE and IgA, respectively, which is mediated by an intrachromosomal recombinational event from the switch (S) region in the Cµ region (Cµ) to one of the downstream S regions. There are many molecules involved in SHM and CSR, such as AID, uracil DNA glycosylase (UNG), mismatch repair (MMR), error-prone DNA polymerases, and nonhomologous end joining (NHEJ). Although some of the enzymes involved have been identified in the last few years, some of the mutational factors have yet to be discovered, and there is still much to be learned regarding how they are coordinated and targeted to the S and V regions.

### Immune regulation

Follicular helper T (TFH) cells have emerged as a new class of immune-regulatory cells specialized to control the stepwise development of antigen-specific B-cell immunity [106–108]. Within the 1st week after priming, antigen-specific TFH cells emerge to initiate antibody secretion [109–111], and GC reaction [112]. In the GC, TFH cells regulate memory B cell development [113–115] and the production of LLPCs [116]. Upon antigen re-challenge, TFH cells promote antigen-specific memory B-cell expansion and the rapid induction of high-affinity PCs [110, 117]. That is, the antigen-specific TFH function is central to multiple facets of B-cell immunity and the formation of LLPCs. Traditionally, the only role of PCs was thought to be antibody secretion [117]. In contrast, a recent study showed that conventional PCs could inhibit the development of TFH cells [118]. By immunizing C56BL/6 mice lacking Blimp-1 in the B-cell compartment to conduct experiments, it was found that the elevation of TFH cells in the absence of PCs provided support for the existence of a negative regulatory loop in vivo. Memory B cells develop in the absence of PCs [119], while their capacity for long-term immune protection is limited in the absence of PCs. IL-21, produced by TFH cells, is central to affinity maturation, GC persistence and function, and B-cell fate determination [119]. Thus, in the absence of PCs, it could be predicted that an increased number of GC TFH cells might result in elevated IL-21 production that could alter normal GC physiology and memory B-cell development and its function. By contrast, constraining the TFH numbers and reducing IL-21 production may progressively increase competitive pressure critical for GC survival signals. In this manner, it might be predicted that the negative regulation of GC TFH function could enhance affinity maturation within the GC and promote memory B cells with higher overall affinities [118]. Therefore, a negative regulatory loop would act as an antigen-specific PC-sensing mechanism to limit the accelerated and explosive PC production that accompanies antigen re-challenge. However, a new point of view has been proposed, namely, that the process of TFH cell development that is primed by LLPCs or DCs participates in the upregulation of surface CD40L, whose downstream signaling pathway is necessary for the function of TFH cells [120]. T cells, primed with DCs or LLPCs, but not with SLPCs, can provide CD40L signals to B cells. Consistent with this, only CD4+ T cells primed with DCs or LLPCs, but not those primed with SLPCs, helped cognate B cells to differentiate to ASCs [121]. Regarding the molecular mechanisms underlying the action of LLPCs, it can be suggested that their effect on TFH polarization might be related to their reduced capacity to prime CD4+ T cells and their reduced IL-2 signaling, which inhibit TFH differentiation by inducing STAT5-dependent expression of Blimp-1, a repressor of BCL-6 [122–124].

## Function of PCs in the intestine

### General characteristics of PCs in the intestine

**Fig. 2:**
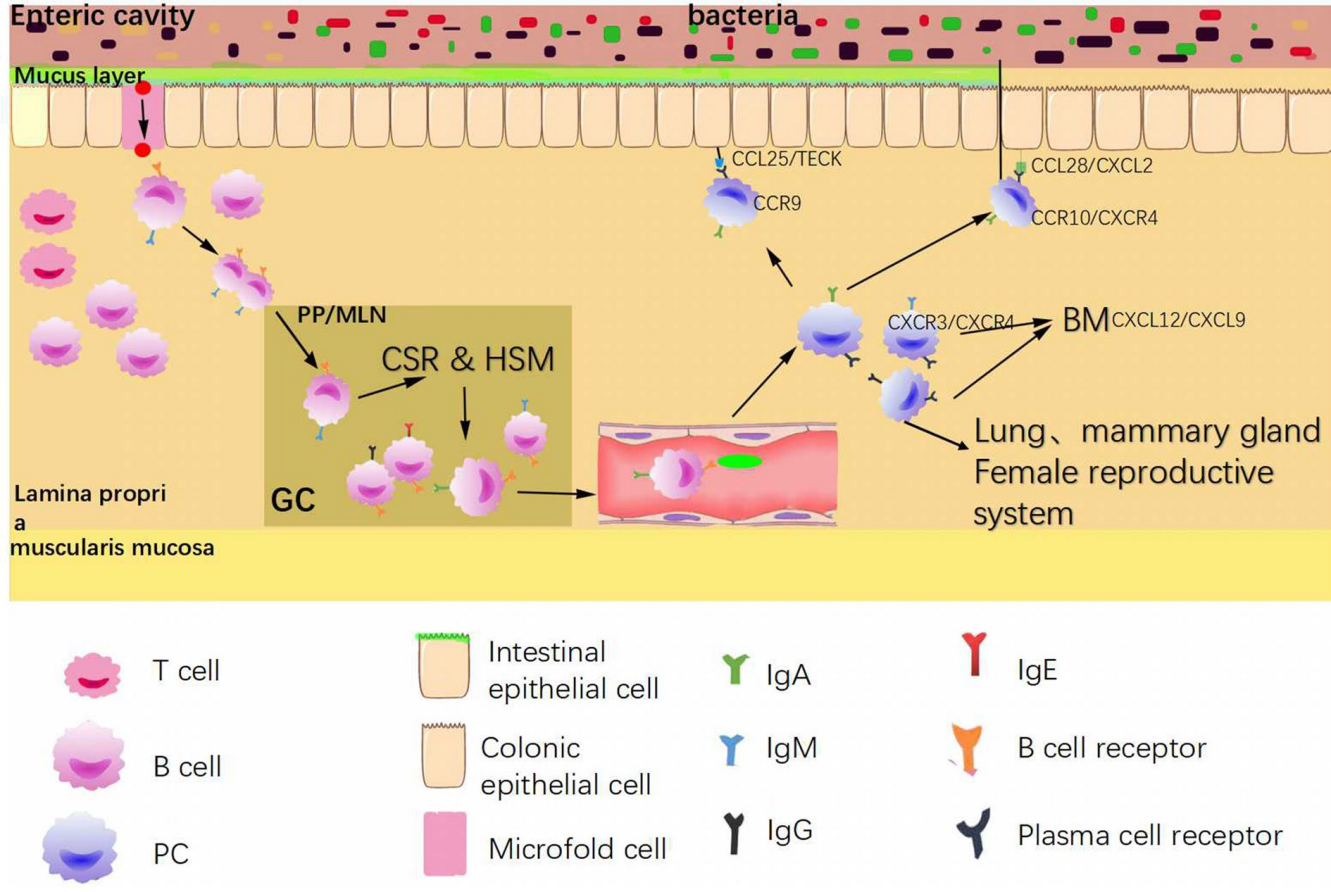
An illustration of the working hypothesis of intestinal PC maturation and metastasis. T and B lymphocytes are abundant in the lymphatic tissues of the gastrointestinal tract. Some of the activated B cells begin to proliferate and generate GCs within PPs or MLNs, where affinity maturation and likely isotype switching from IgM to IgA occur. Most of the fully differentiated B cells leave PPs and MLNs and migrate to the blood. Next, under the action of chemokines, they transfer from the blood circulation to the LP of the small intestine. The IgA PCs express CCR9 and migrate to the small intestine [134], while the expression of CCR10/CXCR4 causes migration to the colon [133]. The IgM and IgG PCs that express CXCR3/CXCR4 migrate to the bone marrow. Lymphocytes from small intestinal lymph nodes return not only to the lamina propria and epithelial cells of the small intestine but also to the lamina propria and epithelial cells of the lungs, mammary gland, and female reproductive system, which is the common mucosal immune system (common mucosal immunologic system, CMIS)

The gut acts as the entryway to many foreign antigens, including food components and potentially harmful pathogens. A first line of defense against these antigens is built by Igs, which can fight against pathogens or toxins [125], an innate barrier of mucus and antimicrobial peptides [126–128]. After the antigen enters the gastrointestinal tract and is combined with the microfold cell (M cell) membrane, the M cell swallows the antigen and forms a pinocytotic vesicle. However, the antigen does not remain in the M cell lysosome; it is immediately transported to the basal membrane of the M cell and then is released into the epithelial lymphoid tissue [129]. There are abundant T and B lymphocytes in the lymphatic tissues of the gastrointestinal tract, but B lymphocytes are the majority [130]. Some of the activated B cells begin to proliferate and generate GCs within PPs or MLNs, which have been identified as locations of affinity maturation and probable isotype switching from IgM to IgA (Fig. 2). However, more investigations have demonstrated that the isotype switch of B220+ IgM+ cells, at least in part, occurs in the LP under the influence of local stimuli [131]. Most of the fully differentiated B cells leave MLNs and PPs and migrate via the lymphatics and thoracic duct into the blood, followed by transfer from blood circulation to the LP of the gut under the action of chemokines.

Lymphocytes from small intestinal lymph nodes return not only to the lamina propria and epithelial cells of the small intestine but also to the lamina propria and epithelial cells of the lungs, mammary gland, and female reproductive system, which is the common mucosal immune system [132]. Based on this concept, the immune function of the intestinal tract can also cause the mucosal protection of other parts of the host, including the respiratory tract and female genital tract. However, because of the various intestinal antigens, will the production of various heterogeneous antibodies promote the occurrence of autoimmune diseases? This issue needs to be explored.

It is common knowledge that IgA ASCs in the mouse small intestine express CCR9, CCR10, and CXCR4 on the cell surface and migrate to their respective ligands CCL25, CCL28, and CXCL12 (also known as stromal cell-derived factor 1) [133] (Fig. 2), and it has been proposed that signaling through CCR9 might be an essential factor that targets cells to the intestine [134, 135]. The CCR9 ligand CCL25/TECK is expressed by epithelial cells of the small intestine but not by those of the colon. However, IgA ASCs in the colon mainly express CCR10 and CXCR4 and migrate to CCL28 and CXCL12. Accordingly, the epithelial cells of the colon are positive for CCL28 and CXCL12 [133]. Murine IgA-producing PCs from the spleen, MLNs and PPs migrate toward CCL25 and CXCL12 [62, 136, 137]. Notably, PCs of the IgG or IgM isotype do not respond to CCL25 but migrate toward CXCL12 and CXCL9 [60, 62, 138], suggesting that the differential expression of chemokine receptors targets PCs to their final destination, depending on the isotype of the Igs.

### Production of the intestinal defender IgA by PCs

**Fig. 3:**
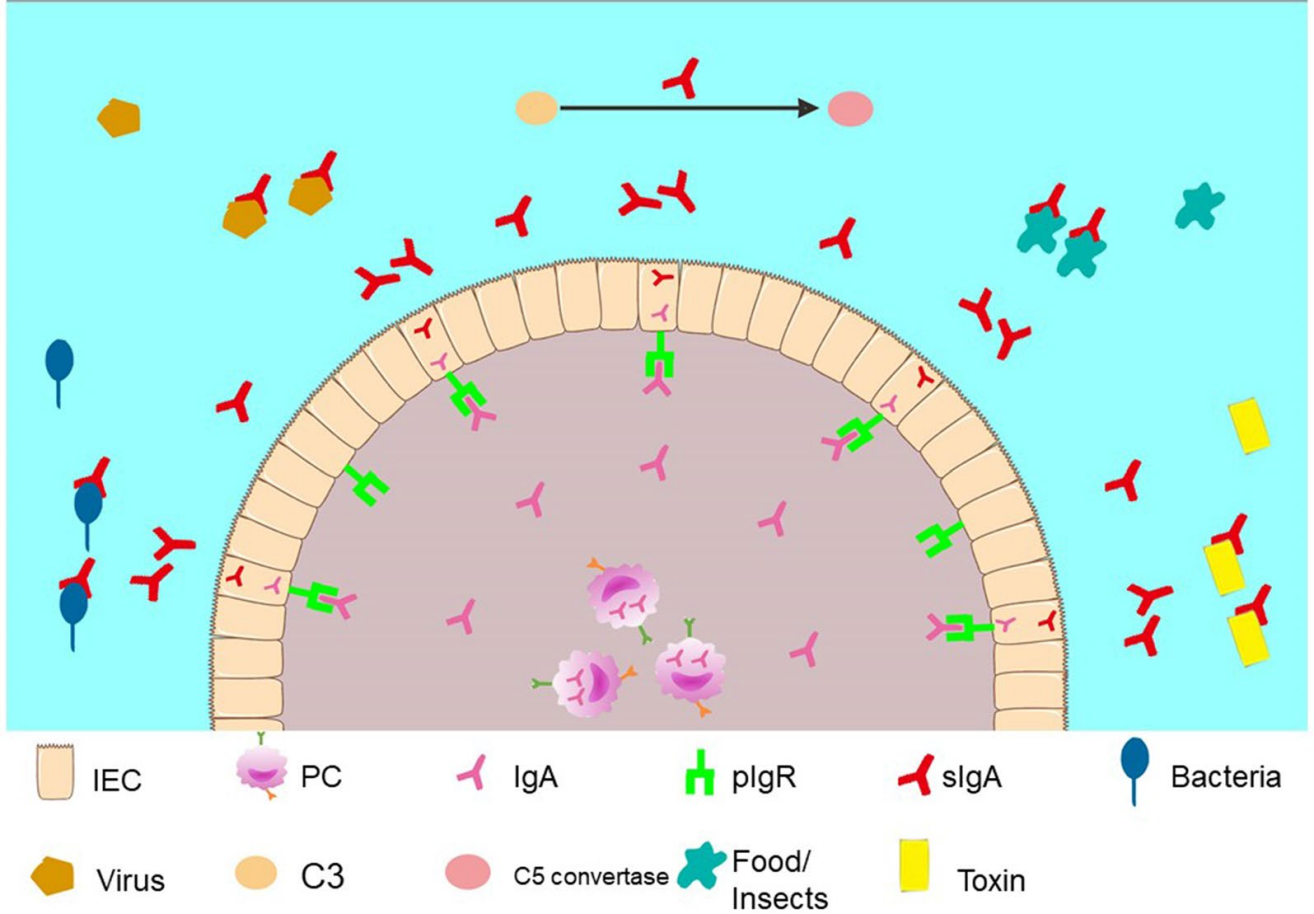
Schematic representation of the function of intestinal sIgA. sIgA in the intestine can not only clear bacteria but also activate the C3 pathway to produce C5 convertase. Additionally, sIgA neutralizes viruses both outside and inside susceptible cells [139, 141] by blocking food antigens and microbial antigens and neutralizing toxins

The humoral immune response in the gastrointestinal tract is mediated by IgA+ memory B cells and IgA-producing PCs in gut-associated lymphoid tissue (GALT). Commensal bacteria, acting as critical stimuli, play a vital role in the maturation of GALT and further induce IgA production by B cells [138]. Class switching to IgA-producing PCs occurs in the PPs and lamina propria, following T-cell-dependent or T-cell-independent mechanisms [139]. Under the stimulation of antigen, IgA-producing PCs then secrete IgA, which binds to polymeric immunoglobulin receptor (pIgR) in intestinal epithelial cells, and the complex is transported to the enteric cavity through intestinal epithelial cells to form secretory IgA (sIgA) accompanied by its immune function (Fig. 3). pIgR, as an IgA-specific receptor with fatal roles in the synthesis, transport and secretion of sIgA, can also protect sIgA from being digested by proteolytic enzymes in the intestine without activation of complement.

sIgA in the enteral cavity can not only inhibit the adsorption of bacteria on the mucosa to clear bacteria [140] but also activate the complement 3(C3) pathway. sIgA in the enteric cavity shows synergistic antibacterial action with complement/alexin and lysozyme [139]. Furthermore, sIgA can neutralize viruses which are outside susceptible cells [139] and viruses that have infected cells [141] (Fig. 3) to establish host–microbial relationships by modulating bacterial epitopes and regulating bacterial metabolism [142]. Although some studies have indicated that sIgA might be specific to individual components of the commensal microbiota [143–146], continuous generation of high-affinity responses against lots of exogenous antigens encountered daily could be complicated in practice. Instead, others have suggested that sIgA has poly-reaction with antibodies which neutralize multiple targets with low affinity to prevent humans from attack of multiple antigens with low “cost” [147–149].

### Regulation of intestinal IgA by multiple factors

sIgA plays a stable role in the regulation of the intestine, while intestinal mucosal lymphocytes show moderate secretion of sIgA. IFN-γ and TNF-α, secreted by gut Th1 cells, downregulate [150] the secretion of IgA, while IL-4, IL-5, IL-6 and IL-10, as well as other cytokines produced by Th2 cells, could induce and enhance secretory component gene expression of IgA. IL-4 is a vital inducer of IgA PCs. IL-5 can increase the response of IgA [151]. Particularly, IL-6 plays a critical role in the orientation, proliferation and differentiation of IgA-secreting PCs at the mucosal effector point [152].

Probiotic bacteria can stimulate systemic and mucosal IgA production in humans [153, 154]. Probiotic bacteria can induce transforming growth factor (TGF)-β, IL-10, and IL-6 expression by mucosal epithelial cells, which potentiate IgA production through B-cell maturation and class switching to IgA [155, 156]. In the process of probiotic-induced intestinal mucosal immune responses, the upregulation of sIgA is based on the following process. First, antigenic substances such as probiotic metabolites or whole cells can enter the PPs through M cells and activate Th2 cells. Next, with the assistance of IL-5 released by CD4+ Th2 cells, IgM+ B cells on PPs convert to IgA+ B cells accompanied by the enhancement of the metabolic product of probiotics with the help of IL-5 released by CD4+ Th2 cells and cytokines such as TGF-β, IL-10, and IL-6.

## PC imbalance and intestinal diseases

PCs play a crucial role in intestinal immune function, and their dysfunction or abnormal secretion of sIgA may often be accompanied by intestinal diseases (see Table 1).

**Table 1:** Relationship between PCs, antibodies and intestinal diseases

IBD	Change in IBD patients	References
B cells, IgA+ and IgG+ PCs	Increased in lesions of IBD patients	MacDermott et al. [164] Brandtzaeg et al. [165]; Conlon and Bird [172]
Mucosal IgG4, serum IgG4 levels serum IgG4/IgG ratios	Increased in IBD patients	Wang et al. [166] Wang et al. [167]

Intestinal food allergies	Intestinal mucosa status	References
Decreased sIgA	Hypersensitivity	Telemo et al. [178] Lilja et al. [177] Thang et al. [179]
Increased IgE	Hypersensitivity	Galli et al. [180] Burton et al. [181]

Intestinal tumors	Impact on tumors	References
Infiltration of B cells in intestine	Progression	Berntsson et al. [182]
Lack of IgA in intestine	Decreased tumor-specific cytotoxicity	Le Gouvello et al. [194] Tosolini et al. [195]

### PCs and IBD

Ulcerative colitis (UC) and Crohn’s disease (CD) fall under idiopathic IBD [157]. UC has a relapsing and remitting course and is characterized by chronic inflammation of the rectum and colon [158, 159]. Similarly, CD has a relapsing–remitting course, mostly occurring in the terminal ileum and right colon.

Circulating B cells have long been identified as CD3 CD19+ B cells [160], including IgD+ CD27− naïve B cells, IgD+ CD27+ non-class-switched memory B cells, and IgD− CD27+ class-switched memory B cells [24, 161]. Upon activation, naïve and memory B cells can differentiate to PCs, which produce antibodies. The numbers of PCs and PBs in UC patients are highly increased compared with those in healthy controls (HCs) [161–163], at the same time B cells and IgA+ or IgG+ PCs are enhanced in the lesions of UC patients [164, 165]. The concentration of IgG, but not that of IgM or IgA, is notably higher in patients than in HCs [166]. Thus, IgG-producing PC infiltration of colonic mucosa leads to chronic inflammation in patients with UC. However, whether its function is pathogenic or protective remains mysterious. Michihide et al. [167] have demonstrated a novel aspect of UC pathogenesis in which unique IgG PCs infiltrate the inflamed mucosa via CXCR4 and critically influence UC pathogenesis by exacerbating mucosal inflammation through the activation of ‘pathogenic’ intestinal CD14 macrophages via IgG-IC FcγR signaling. Additionally, the concentration of serum IgG has been positively associated with the numbers of CD138+ CD38+ CD20− CD19+ and IgG+ CD38+ CD20− CD19+ PBs in patients with UC. A case–control study by Wang found that patients with IBD had a dramatically higher mucosal IgG4 than healthy individuals, and in patients with UC, mucosal IgG4 was positively correlated with serum IgG4 level and serum IgG4/IgG ratio [168]. The characterization of these cells may provide a complementary approach to monitoring the disease activity or therapeutic efficacy in active UC patients, and high IgG4 level may be a biomarker for a new subset of IBD. Therefore, these discoveries perhaps provide a new method of controlling IBD by the specific inhibition of PCs that produce IgG antibodies. It will be worthwhile to explore further whether the precise attack on specific antibodies can reduce the side effects of immunosuppressive drugs in the treatment of the disease.

Of course, there is a novel viewpoint on the pathogenesis of IBD yet. Epidemiological surveys [169–171] have found that, with the improvement of people’s living standards, the incidence of IBD has been increasing, and the incidence in the urban population is larger than that in the rural population. Some scientific research has suggested this phenomenon results from the improvement of living standards and changes in diet to foods with high energy density, animal proteins, total and saturated fats, sugar, and salt but low levels of plant-derived fibers. A “Western” diet may break the balanced composition of the gut microbiome, leading to perturbed immune responses, including effects on B-cell production, activation and maturation [171, 172]. Increased autoreactive and pro-inflammatory antibodies have been confirmed in obese humans and high-fat diet (HFD)-fed mice [173–175], The increased natural autoreactive IgM antibodies under an HFD form an immune complex with apoptosis inhibitors of macrophages, which promotes IgG autoantibody production contributing to the occurrence of IBD [176].

### PCs and intestinal food allergies

Food allergies, as a type of immune-mediated reaction to ingested food proteins, have become a serious public health issue and harm the health of children and adults with increasing incidence yearly. In food allergic reactions, the intestinal mucosal immune system plays a momentous role in the process of identifying and removing pathogenic bacteria and inducing the body to form immune tolerance. sIgA on the surface of the mucosa recognizes certain antigen substances from food or air so that these antigens are dissociated from the surface of the mucosa and are not allowed to enter the body, thus avoiding the systemic immune response [177]. Oral tolerance is due to this function of sIgA [178]. Antibodies to food proteins appear in the serum of some patients, resulting in an increased incidence of hypersensitivity, which may be related to the lack of sIgA that weakens the ability to prevent the food antigens from invading the intestinal mucosa. Some studies of infants have shown that whey protein allergies are generally related to local IgA response defects and IgE-mediated hypersensitivity. Thang et al. [179] exposed β-globulin-sensitized mice to probiotics, and the results showed that the inhibition mechanism of the allergic reaction was the increase in sIgA in the intestinal tract, which prevented the invasion of food allergens in the intestinal mucosa to inhibit allergic reactions. The occurrence of intestinal food allergies is associated with a reduction in sIgA. Titers of high-affinity allergen-specific IgE antibodies are increased in patients with symptomatic allergy [180]. Allergen-specific IgE has also been suggested to lead to allergy pathogenesis by promoting antigen presentation and epitope spreading by the uptake of antigen-IgE complexes through low-affinity CD23, an IgE receptor, present on antigen-presenting cells (APCs) [181]. Thus, could increasing the contents of IgA and IgE on PCs in the local colon be a new strategy to treat irritable bowel disease? Does it cause immune dysfunction and autoimmune diseases? These problems are worthy of further exploration.

### PCs and intestinal tumors

Colorectal cancer (CRC) is one of the most common tumor types worldwide. There are many risk factors that lead to CRC, and one of the most important factors is chronic intestinal inflammation. A prospective population-based cohort showed that B cell-specific CD20, CD138, and immunoglobulin kappa C expression correlated significantly with lower tumor stage it is also demonstrated that infiltration of B cells in CRC had a great impact on tumor progression and prognosis [182]. A recent study of immunized CRC patients showed that patients with higher IgA titers showed better survival, although the relationship was only significant in univariate regression analyses [183]. Several studies in vitro also indicated that IgA can mediate tumor-specific cytotoxicity through NK cells or polymorphonuclear phagocytes [183–186]. CCL28 signaling through CCR10, together with a4b7-MAdCAM-1 interactions, is a pivotal part of IgA-producing PB migration to intestinal sites, both in the steady state and during vaccine-induced immune responses [187–190]. However Muthuswamy et al. recently argued persuasively [191] that the endothelium in colon tumors expresses less MAdCAM-1 than the surrounding unaffected mucosa. More research has shown that CCL28 production is also reduced in colon tumors [192, 193]. The lower the IgA secretion is at the tumor site, the higher is the risk of bacterial penetration into the tissues leading to reduced local barrier function [194, 195]. Above all, IgA may reduce bacteria-induced inflammation at the tumor site, and a lack of IgA locally at the tumor site may lead to increased local inflammation and drive tumor progression.

## Conclusion

To summarize the above arguments, PCs with their various antibodies act as warriors in humoral immunity. In healthy intestinal mucosa, PCs play an essential role in maintaining homeostasis by producing many antibodies, especially sIgA. As an intestinal defender, sIgA plays a stable role in the regulation of the gastrointestinal neuroendocrine-immune system network. Notably, once the regulation of IgA-secreting cells or sIgA is imbalanced, such as over- or under-activation, diseases such as IBD, intestinal food allergies, and tumors may generate. New findings concerning intestinal PCs and IBD, as well as tumors, can be very helpful for studies and disease treatments. Meanwhile, there are many details awaiting clarification, as well as many unresolved issues.

## Abbreviations

PCs: plasma cells
SLPCs: short-lived plasma cells
LLPCs: long-lived plasma cells
IBD: inflammatory bowel disease
TLR: toll-like receptor
LP-DCs: lamina propria dendritic cells
PPs: Peyer’s patches
MLNs: mesenteric lymph nodes
BM: bone marrow
NGFR/TNFR: nerve growth factor receptor/tumor necrosis factor receptor
BLIMP-1: B lymphocyte-induced maturation protein-1
IRF4: interferon regulatory factor 4
XBP-1: X-box binding protein-1
GC: germinal center
ERK/MAPK: MAP kinase/mitogen-activated protein kinase
CSR: class switch transformation
PERK: (PKR)-like ER kinase
ATF6a: activating transcription factor 6
IRE1: inositol-requiring enzyme-1
APRIL: proliferation-inducing ligand
MSCs: mesenchymal stem cells
VEGF: vascular endothelial growth factor
GPCR: G-protein-coupled receptors
SHM: somatic hypermutation
TFH: follicular helper T
GALT: gut-associated lymphoid tissue
UC: ulcerative colitis
CD: Crohn’s disease
HFD: high-fat diet
APCs: antigen-presenting cells
CRC: colorectal cancer
